# Effect of aerobic exercise training and cognitive behavioural therapy on reduction of chronic fatigue in patients with facioscapulohumeral dystrophy: protocol of the FACTS-2-FSHD trial

**DOI:** 10.1186/1471-2377-10-56

**Published:** 2010-06-30

**Authors:** Nicoline BM Voet, Gijs Bleijenberg, George W Padberg, Baziel GM van Engelen, Alexander CH Geurts

**Affiliations:** 1 Nijmegen Centre for Evidence Based Practice; Department of Rehabilitation, Radboud University Nijmegen Medical Centre; Nijmegen, The Netherlands; 2 Expert Centre Chronic Fatigue, Radboud University Nijmegen Medical Centre; Nijmegen, The Netherlands; 3 Department of Neurology, Donders Centre for Neuroscience; Radboud University Nijmegen Medical Centre; Nijmegen, The Netherlands

## Abstract

**Background:**

In facioscapulohumeral dystrophy (FSHD) muscle function is impaired and declines over time. Currently there is no effective treatment available to slow down this decline. We have previously reported that loss of muscle strength contributes to chronic fatigue through a decreased level of physical activity, while fatigue and physical inactivity both determine loss of societal participation. To decrease chronic fatigue, two distinctly different therapeutic approaches can be proposed: aerobic exercise training (AET) to improve physical capacity and cognitive behavioural therapy (CBT) to stimulate an active life-style yet avoiding excessive physical strain. The primary aim of the FACTS-2-FSHD (acronym for Fitness And Cognitive behavioural TherapieS/for Fatigue and ACTivitieS in FSHD) trial is to study the effect of AET and CBT on the reduction of chronic fatigue as assessed with the Checklist Individual Strength subscale fatigue (CIS-fatigue) in patients with FSHD. Additionally, possible working mechanisms and the effects on various secondary outcome measures at all levels of the International Classification of Functioning, Disability and Health (ICF) are evaluated.

**Methods/Design:**

A multi-centre, assessor-blinded, randomized controlled trial is conducted. A sample of 75 FSHD patients with severe chronic fatigue (CIS-fatigue ≥ 35) will be recruited and randomized to one of three groups: (1) AET + usual care, (2) CBT + usual care or (3) usual care alone, which consists of no therapy at all or occasional (conventional) physical therapy. After an intervention period of 16 weeks and a follow-up of 3 months, the third (control) group will as yet be randomized to either AET or CBT (approximately 7 months after inclusion). Outcomes will be assessed at baseline, immediately post intervention and at 3 and 6 months follow up.

**Discussion:**

The FACTS-2-FSHD study is the first theory-based randomized clinical trial which evaluates the effect and the maintenance of effects of AET and CBT on the reduction of chronic fatigue in patients with FSHD. The interventions are based on a theoretical model of chronic fatigue in patients with FSHD. The study will provide a unique set of data with which the relationships between outcome measures at all levels of the ICF could be assessed.

**Trial registration:**

Dutch Trial Register, NTR1447.

## Background

Facioscapulohumeral dystrophy (FSHD) is the third most common inherited neuromuscular disorder. It is an autosomal dominant slowly progressive myopathy with a variable age of onset, mostly in the second or third decade of life. Its yearly incidence rate is approximately 1:20.000 [1]. The disease primarily affects the facial muscles, the muscles of the shoulder girdle (most typically the scapula stabilizers) and various leg muscles, while pelvic and trunk muscles are eventually affected as well [2-4]. The pattern of muscle weakness is often asymmetrical, and the rate and extent of progression may vary considerably with sudden periods of unexplained rapid disease progression. In a small percentage of the patients, even respiratory insufficiency may occur [5]. Only very recently, evidence became available that there may be a selective involvement of the central nervous system as well, in terms of decreased grey matter volume [6] and reduced intracortical inhibition [7]. Although FSHD is associated with a partial deletion of a critical number of repetitive elements (D4Z4) on chromosome 4q35, to date no causal gene has been identified and no curative treatment is available [3,8]. FSHD may eventually lead to serious disabilities of speech, swallowing, reaching, standing and walking, even in early adulthood. Twenty percent of the patients become wheelchair bound. Since no cure is available, rehabilitation is the mainstay of treatment [2,3,9].

**Figure 1:**
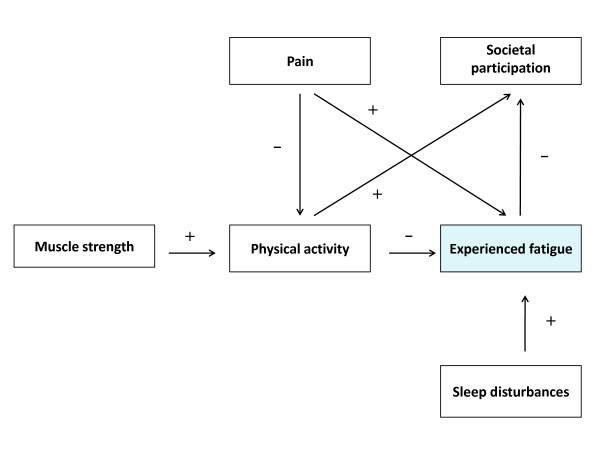
**Model of perpetuating factors of experienced fatigue in patients with FSHD**.

Only recently it was shown by our group that severe fatigue, defined as a score equal or higher than 35 on the subscale fatigue of the Checklist Individual Strength (CIS-fatigue), was reported by 61% of the patients with FSHD. These severely fatigued patients had more problems with physical and social functioning as well as with mental and general health than similar patients without a severe fatigue. They also had more problems with concentration, initiating and planning [10]. As such, experienced fatigue should be clearly distinguished from muscle weakness, which is probably the most common and characteristic symptom of FSHD [11]. In a longitudinal study, we built a model of perpetuating factors for fatigue in patients with FSHD (figure 1). It appeared that lack of physical activity, sleep disturbances and pain all contributed to experienced fatigue. In addition, loss of muscle strength and pain contributed to fatigue through a lower level of physical activity. Ultimately, experienced fatigue and physical inactivity both contributed to the level of societal participation [12]. Thus, theoretically, in order to improve societal participation one should improve muscle strength, reduce pain, optimize physical activity and alleviate experienced fatigue. In addition, falling appears to be a major problem among FSHD patients. Our group was able to show that 65% of the patients reported falling at least once a year [13]. Since fall incidents often lead to fear of falling and avoidance behaviour, they have a serious negative impact on physical activity and participation.

Improving muscle strength by strength training and/or (anabolic) medication has shown not to be successful in patients with FSHD [14]. Until now, only one trial has investigated low-intensity aerobic exercises, indicating that aerobic training is a safe method to increase exercise performance [14,15]. Although, in general, physical activity does not appear harmful [16,17], more research is needed to establish whether AET is beneficial in patients with FSHD. Besides improving physical (aerobic) capacity, it seems important to optimize physical activity and change behaviour in daily life. Indeed, symptoms and signs of muscle weakness and fatigue as well as the anticipation of a (further) decline in physical capacity may elicit an inactive life-style, which may disproportionally affect physical activity, fatigue and societal participation. From this perspective, it might be beneficial to alter illness cognitions and coping styles by means of CBT. However, evidence for the effectiveness of CBT in patients FSHD is not yet available.

The primary objective of the FACTS-2-FSHD trial is:

- to study the efficacy of AET and CBT for decreasing chronic fatigue in patients with FSHD. It is hypothesized that both AET and CBT are more effective in decreasing fatigue than usual care, which is no therapy at all or occasional (conventional) physical therapy. The improvement by AET may be obtained through enhancement of physical (aerobic) capacity, whereas beneficial effects of CBT may be achieved through changes in daily activities and behaviour. By changing illness cognitions and improving coping style, the balance between actual behaviour and physical capacity will be optimized. Since changes achieved by CBT are more 'intrinsic', possible beneficial effects of CBT may last longer than those of AET.

Secondary objectives of the FACTS-2-FSHD trial are:

- to evaluate the effects of AET and CBT on bodily functions and structures as defined by the International Classification of Functioning, Disability and Health (ICF): lower extremity muscle strength, pain, psychological well being, cardiovascular risk factors, aerobic exercise tolerance, sleeping pattern, as well as biomarkers in blood and urine and structural and metabolic muscle tissue characteristics.

- to evaluate the effects of AET and CBT on the ICF level of activities: physical activity in daily life, self perceived functional status, and fall incidence.

- to evaluate the effects of AET and CBT on the ICF level of participation: limitations in participation and autonomy and quality of life.

- to evaluate the effects of AET and CBT on environmental and personal factors as defined by the ICF: coping style, illness cognitions, concentration problems, motivation, caregiver strain, experienced fatigue of the caregiver, social support and coping of the caregiver.

## Methods

### Study population

It is intended to include 75 FSHD patients, diagnosed on both clinical and genetic grounds, aged 18 years and older. All patients who participated in previous FSHD studies at the Radboud University Nijmegen Medical Centre [10,12,14,18] are approached by the primary investigator (NV). In addition, all patients known at the departments of Neurology and Rehabilitation of the Radboud University Nijmegen Medical Centre, University Medical Centre of Utrecht, Amsterdam University Medical Centre or any of the affiliated rehabilitation centres are invited to participate as well. In addition, patients who are registered in the Dutch neuromuscular "computer registry of all myopathies and polyneuropathies" (CRAMP) database [19] and/or who are member of the Dutch patient support organisation "vereniging spierziekten Nederland" (VSN) will be invited by the primary investigator (NV) and a member of the VSN, respectively, to take part in the study. If the patient is willing to participate, the primary investigator (NV) will check the inclusion and exclusion criteria (table 1) and estimate the disease severity using the Ricci score [20]. When a patient meets all the criteria, oral and written informed consent are obtained according to the declaration of Helsinki. Separate consent is asked to (i) obtain blood and urine samples, and/or (ii) undergo magnetic resonance imaging (MRI)/spectroscopy (MRS), and/or (iii) undergo muscle ultrasound. The study protocol was approved by the Dutch ethical committee CCMO (Centrale commissie mensgebonden onderzoek) and all participating centers granted (ethical) approval to participate.

**Table 1:** Inclusion and exclusion criteria

Inclusion criteria
(1) age 18 years and older

(2) suffering from severe fatigue (CIS-fatigue ≥ 35) [21]

(3) ability to walk independently (ankle-foot orthoses and canes are accepted)

(4) being able to exercise on a bicycle ergometer

(5) being able to complete either type of intervention

**Exclusion criteria**

(1) cognitive impairment

(2) insufficient mastery of the Dutch language

(3) neurological or orthopedic co-morbidity interfering with the interventions or possibly influencing outcomes

(4) pregnancy

(5) use of psychotropic drugs (except simple sleeping medication)

(6) severe cardiopulmonary disease (chest pain, arrhythmia, pacemaker, cardiac surgery, severe exertional dyspnea, emphysema)

(7) epileptic seizures

(8) poorly regulated diabetes mellitus or hypertension

(9) clinical depression, as diagnosed with the Beck Depression Inventory for primary care (BDI-PC) [22,23]

### Randomization and blinding

**Figure 2:**
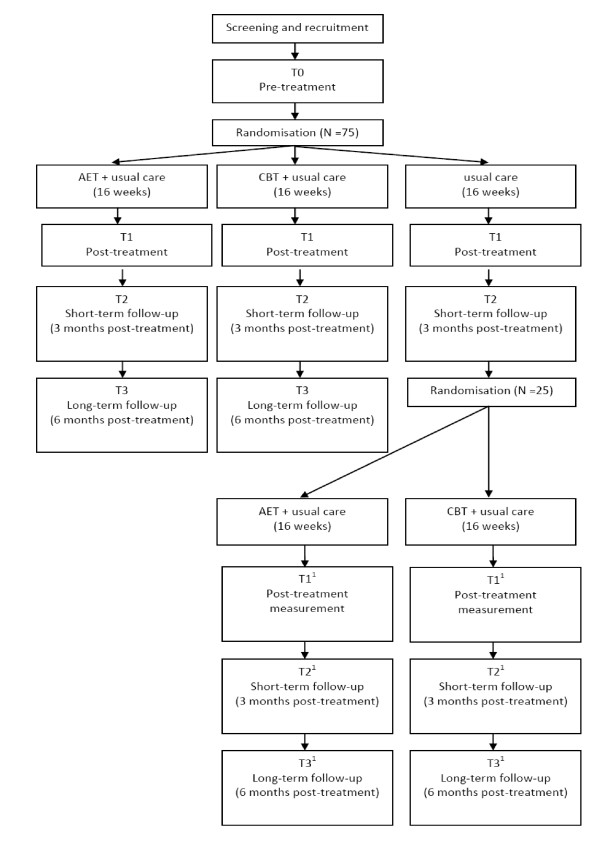
**Study design T**.

Participants fulfilling all inclusion and exclusion criteria are randomised to one of three groups by creating computer-generated 'blocks' ensuring that the same number of participants is allocated to each group. Experimental group 1 (E1) receives AET and usual care for 3 times a week during 16 weeks. Experimental group 2 (E2) receives CBT and usual care once time a week during 16 weeks. Thereafter, both groups are followed up for 6 months. A third group (C) receives usual care only for 16 weeks and serves as a waiting list control group. After another 3-months follow-up (i.e. 7 months after inclusion), this group will as yet be randomised to either AET or CBT for 16 weeks and followed up until 6 months later (total time in study 17 months) (figure 2). All outcomes are assessed by blinded and independent physical therapists. At the beginning of each assessment, patients are always instructed not to reveal their group allocation to the blinded assessor.

### Interventions

#### Usual care

All participants receive usual care. In the Netherlands, patients with FSHD typically receive no therapy at all, or occasional (conventional) physical therapy. Patients are not restricted in any activities, but all co-interventions are monitored throughout the study by diaries and at every measurement.

#### Aerobic Exercise Training (AET)

The AET consists of aerobic cycling exercise on a bicycle ergometer. The training program has a duration of 16 weeks and comprises home training twice a week and a supervised training once a week. Training sessions consist of a 30-minute aerobic exercise period with a warming-up and cooling-down period of 5 and 3 minutes, respectively. The cardiovascular load during the training period is individually adjusted and increased from a level of 60% to 75% of the heart rate reserve (HRR). HRR is the difference between the predicted maximum heart rate and the measured resting heart rate. The HRR is equivalent to the difference between the maximum and resting maximal oxygen consumption (VO2max). Each participant is learned how to adjust the physical load to the preferred individual heart rate. Participants are supplied with a Monark 827E bicycle ergometer, a Garmin forerunner 50 heart rate watch with breast belt, and a log book with training instructions at home for the duration of the intervention. During each training, the heart rate is monitored continuously by the breast belt. The number of training sessions, the total time spent on AET, possible adverse effects and the training parameters (physical load, heart rate) are recorded in the individual log book. Once a week, individually supervised training is given by trained physical therapists during one-hour sessions in small groups in a nearby rehabilitation centre. During these sessions, therapy compliance in the home situation is verified by reading out the heart rate watches and checking the log books. In addition, instructions for the next week are provided. The (unblinded) primary investigator and physician (NV) gives instructions to the physical therapists and performs integrity checks at each treatment location.

#### Cognitive Behavioural Therapy (CBT)

CBT will be focused on the perpetuating factors of fatigue as established in previous research [10,12,24] and based on experience in clinical practice. These factors encompass insufficient coping with the disease, dysfunctional illness cognitions, catastrophizing, deregulation of sleep, deregulation of activity, low social support and negative social interactions (see appendix 1 for the various modules). Because of large inter-individual differences, CBT will be adapted to the needs of each patient. For instance, barriers to become more physically active are explored and possibly alleviated in some patients, whereas overactivity is reduced in others. To determine which modules are appropriate, each perpetuating factor is assessed with specific tests, and within each module, the CBT approach is standardized (see appendix 1). The precise number of sessions is dependent on the number of modules. Each session has a duration of one hour and is given at the most nearby participating centre by a registered cognitive behavioural therapist, especially trained in CBT for FSHD. The therapists have been specifically trained to use the diagnostic tests and indicate the different modules and are regularly supervised by one of the investigators (GB).

#### Compliance and attrition

Therapy compliance is assessed by recording the number of treatment sessions (AET and CBT). For the participants randomized to AET, the total time spent on the bicycle ergometer at home is recorded as well. When applicable, participants are asked for their reasons for poor compliance. In the case of therapy drop out, patients are asked for the reason of non compliance and are stimulated to continue participation in the assessments until the last follow-up.

#### Outcome assessment

Outcome measures are listed in table 2. The primary outcome measure is fatigue severity as assessed with the CIS-fatigue [21]. Secondary outcome measures are categorized according to the different ICF levels [25]. Outcome measurements are obtained at the Radboud University Nijmegen Medical Centre at the start of the study period (T0), immediately after the intervention period of 16 weeks (T1) and after 3 (T2) and 6 months of follow-up (T3). Observers of the secondary outcome measurements are experienced physical therapists blinded for treatment allocation. At the first measurement (T0), demographic data is obtained by the primary investigator and physician (NV), as well as a general and FSHD related medical history, anthropometric measures (diastolic and systolic blood pressure in mm, resting pulse rate in beats per minute and auscultation of heart and lungs), to verify eligibility. The baseline (T0) and post treatment (T1) visits consist of muscle strength testing of the thigh and aerobic exercise tolerance testing. Participants are asked to complete the questionnaires and to wear the actometer for 12 consecutive days. An actometer is a motion sensing device that can register and quantify human physical activity and has to be worn at the ankle [26,27]. Blood and urine analyses and MRI, MRS and ultrasound measurements of the thigh muscles are performed. Follow-up measurements (T2 and T3) consist of muscle strength testing of the thigh, aerobic exercise tolerance testing, questionnaires are completed and the actometer is provided.

**Table 2:** Outcome measures and tests

	Tests
**Primary outcome measure**	

Fatigue severity	Checklist Individual Strength (CIS subscale fatigue) [30]

**Secondary outcome measures**	

***ICF: bodily funtions***	
Aerobic exercise tolerance	Maximal oxygen consumption (VO2 max) using the Astrand protocol[31]
	6-minutes walking test [32]
	Resting heart rate
Muscle strength of quadriceps,	Quantitative Muscle Assessment using fixed myometry testing
hamstrings and tibialis anterior	system (QMA) [18]
muscles	
Cardiovascular risk factors	Blood pressure*
	Abdominal circumference
	Weight/Body Mass Index (BMI)
	Percentage body fat
	Visual Analogue Scale (VAS) [33]
Pain	Daily Observed Pain (during a period of 2 weeks) [27,34]
Psychological well-being and	Brief Symptom Inventory (BSI) [35]
sleeping pattern	
Metabolic biomarkers	Blood and urine analyses for creatine, glucose, creatine kinase,
	sodium, potassium, calcium, phosphorus, ureum, ALAT, ASAT,
	gamma-GT, bilirubine, AF and LDH***
Structural and metabolic muscle characteristics	T1 MRI, T2 MRI, ^31^P and ^1^H MRS analysis of muscle specific creatine, extramyocellular lipids, intramyocellular lipids, levels and phosphometabolites in thigh muscles; ultrasound of thigh muscles

***ICF: activities***	
Physical activity in daily life	Actometer, a motion sensing device (during a period of 2 weeks)
	[26,27]
	Daily Observed Spontaneous physical activity (during a period of 2 weeks) [27,34].
	Checklist Individual Strength (CIS subscale physical activity) [30]
Self perceived functional status	Sickness Impact Profile (SIP subscales mobility control and mobility range, social behavior) [36]

***ICF: participation***	
Limitations in participation and autonomy Quality of life Fall incidence	The Impact on Participation and Autonomy Questionnaire (IPAQ)[37]
	36-Item Short-Form Health Survey (SF-36) [38]
	Telephone computer (weekly calls)**

***ICF: personal factors***	
Coping	Coping Inventory for Stressful Situation (CISS) [30]
	ALCOS-16 [39]
Illness cognitions	Ziekte cognitie lijst (ZCL) [40]
Concentration problems	Checklist Individual Strength (CIS subscale concentration) [21]
Motivation	Checklist Individual Strength (CIS subscale motivation)[21]

***ICF: environmental factors***	
Caregiver strain	Caregiver Strain Index of partner/caregiver (CSI) [41]
Experienced fatigue of patient	Checklist Individual Strength (CIS subscale fatigue, filled in by
from perspective of relative.	relative about patient) [21]
Experienced fatigue of partner.	Checklist Individual Strength (CIS subscale fatigue, filled in by relative about him/herself) [21]
Social support	Sociale steunlijst-subschaal discrepantie (SSL-D verkort) [42]
Coping of partner	Coping Inventory for Stressful Situations (CISS) [30]

*Measured only at inclusion. **Participants will be called weekly throughout the study by a telephone-computer to obtain fall incidence; in the case of fall incident(s), a subsequent telephone interview is held to obtain information about the cause and circumstances of falling, fall direction, possible injury and ability to get up from the floor. ***In all groups, three extra urine samples will be obtained at week 4, 8 and 12 of the intervention period.

At the level of bodily functions and structures, patients are asked to give separate consent for several 'invasive' assessments at baseline and after the intervention period. Blood and urine samples are collected by experienced nurses and will be explored by nuclear magnetic resonance (NMR) for possible biomarkers of disease and response to the interventions. Ultrasound measurements of the thigh muscles are made by an experienced ultrasound professional, blinded for the treatment allocation, and analyzed for muscle thickness and echo intensity. In addition, Magnetic Resonance Imaging (MRI) and ^31^P and proton (^1^H) Magnetic Resonance Spectroscopy (MRS) are performed by trained professionals, blinded for the assignment to the intervention. It has been shown that in vivo MRS is able to produce spectra of multiple metabolites simultaneously and is well suited to study energy metabolism in patients with muscular dystrophies [28,29]. The MR examinations start with T1 and T2 weighted images of the thigh for detailed structural analysis. Muscle involvement is specifically assessed by the presence of fatty infiltration on T1 weighted MR images. ^1^H MRS is used to assess muscle specific creatine as well as extramyocellular lipids and intramyocellular lipids levels, whereas ^31^P MRS is applied to get information about tissue pH and the level of high energy phosphates present in the different thigh muscles.

#### Adverse events

An adverse event is defined as any undesirable experience or outcome. Specially assigned site investigators are instructed to report all adverse events immediately to the primary investigator (NV) and to evaluate each event for its date of onset, possible relation to the interventions (based on clinical judgment), possible treatment and course in time. In addition, adverse events can be reported by the participants directly to the primary investigator and physician (NV). All adverse events reported will be carefully monitored and registered until they have abated or a stable situation has been reached.

#### Statistical Analysis

Generalized estimated equations analysis will be used to investigate differences in the effects on primary and secondary outcome measures between the study groups and to investigate the influence of possible effect modifiers. When necessary, analyses will be adjusted for group differences in fatigue severity and physical activity at baseline. Data will be analyzed according to the intention-to-treat principle.

#### Power

In order to detect a 10% group difference (E1 and E2 versus C) in change in fatigue severity between the start and the end of the intervention period (assuming difference in standard deviation between the start and the end of the intervention (SDdif) = 10%, α = .05, β = .80), 20 participants per group are required. With an expected drop-out rate of maximally 25%, 25 participants will be recruited in each group (n = 75).

## Discussion

To the best of our knowledge, the FACTS-2-FSHD study is the first randomized clinical trial which evaluates the effect of AET and CBT on the reduction of chronic fatigue in patients with FSHD.

This study has several strengths. First, the selected interventions are based on a theoretical model of chronic fatigue in patients with FSHD [12] and are compared with usual care in a randomized design. Until now, only one randomized controlled trial has been conducted that could not establish a beneficial effect of muscle strength training compared to no training in FSHD [14,18]. In addition, one trial has been conducted that investigated low-intensity aerobic exercises in FSHD. Although this latter study reported improved maximal oxygen uptake and workload as a result of training, this was an uncontrolled and unblinded trial of only 8 patients [15]. The majority of the training studies in patients with muscle disorders did not include a (no-training) control group or used healthy subjects as controls. In addition, data are often presented for mixed groups of muscle disorders [17]. Second, the proposed study uses a broad arsenal of secondary outcome measures at all levels of the ICF, including 'invasive' measurements of possible biomarkers in blood and urine as well as measurements of structural and metabolic muscle characteristics. This approach will provide a unique set of data with which it should be possible to accurately assess the relationships between disease characteristics, loss of bodily functions, activity limitations and restrictions in societal participation in patients with FSHD. Third, all patients will be followed up until 6 months after the interventions, which will not only provide information about the maintenance of effects, but also about any long-term adverse events.

A limitation of this study is that the sample size calculation was based on detection of a 10% difference between the intervention groups and the control group, presuming more or less equal effect sizes of AET and CBT. Detecting more subtle differences in the effectiveness between both interventions would require a much larger sample size. In the Netherlands alone, such a trial would not be feasible.

In conclusion, the FACTS-2-FSHD study will increase our insight into the effectiveness of aerobic exercise training and cognitive behavioural therapy to reduce chronic fatigue and to optimize physical activity and capacity in patients with FSHD. A successful outcome of this study has the potential to change existing (inter)national guidelines for physical training and to improve the quality of life in patients with FSHD.

## Abbreviations

^1^H: proton; ^31^P: phosphorous; AET: aerobic exercise therapy; CBT: cognitive behavioural therapy; CCMO: Centrale commissie mensgebonden onderzoek; CIS: Checklist individual strength; CRAMP: computer registry of all myopathies and polyneuropathies; E1: experimental group 1; E2: experimental group 2; FACTS-2-FSHD: acronym for Fitness And Cognitive behavioural TherapieS/for Fatigue and ACTivitieS in FSHD; FSHD: facioscapulohumeral dystrophy; HRR: heart rate reserve; ICF: International Classification of Functioning, Disability and Health; MRI: magnetic resonance imaging; MRS: magnetic resonance spectroscopy; NMR: nuclear magnetic resonance; VO2max: maximal oxygen consumption; VSN: Vereniging spierziekten Nederland.

## Competing interests

The authors declare that they have no competing interests.

## Authors' contributions

NV is primary investigator and responsible for data collection and analysis and for drafting the manuscript. AG, BvE, GB, and GP designed and supervised the study. AG and BvE obtained funding for the study. All authors helped in finalizing the manuscript.

## Appendix 1 Different modules of Cognitive Behavioural Therapy

### Perpetuating factors: insufficient coping with the disease

Insufficient coping with the disease is assessed with the Impact of event Scale [43,44]. A participant can continue to be occupied with the period of being diagnosed with FSHD. By means of talking or writing about this experience (which can be referred to as 'exposure'), the participant will acquire better coping skills. Fear of progression is assessed with a questionnaire especially designed for FSHD. The therapist helps the participant to formulate explicit words to describe the thoughts of fear of progression. These thoughts are challenged against reality (reality testing). In this way, daily unhelpful thoughts about the disease progression are reduced and put into perspective.

### Perpetuating factors: d*y*sfunctional cognitions regarding fatigue, activity and other symptoms

Dysfunctional cognitions relate to a variety of ideas, including a participant's idea of lack of control over symptoms, and dysfunctional cognitions about symptoms, such as catastrophizing. The sense of control in relation to fatigue complaints *will be assessed with the *self-efficacy scale [34,45].

### Perpetuating factors: catastrophizing

Catastrophizing will be assessed with the Jacobsen Fatigue Catastrophizing Scale [46]. These cognitions are disputed and more helpful ways of thinking are taught.

### Perpetuating factors: deregulation of sleep

Deregulation of sleep is based on self-report in a sleep diary [47]. An irregular sleep-wake rhythm can perpetuate fatigue. To restore the biologic rhythm, participants are encouraged to adhere to fixed bedtimes and wake-up times and discouraged from sleeping during the day, or they are helped with adapting fixed rest period(s).

### Perpetuating factors: *deregulation of activity*

Deregulation of activity is based on activity (stepping) monitoring using an actometer and a physical activity questionnaire *(*Physical Activity Scale for Individuals with Physical Disabilities [44,48]). Some patients experience fluctuating periods of activity with subsequent periods of rest during a longer period. Others avoid activity because they are concerned that activity increases fatigue; consequently, they are physically inactive. For patients with fluctuating activity levels, a base level should be established by alternating rest and activities to prevent bursts of activity. Once the participant has set a base level, the physical activity program is started, usually twice a day, starting with 5 to 10 minutes of an activity such as walking or cycling. The activity is increased by 1 minute a day each time the activity is performed (ending at a maximum of 2 times 60 minutes minutes per day). The inactive participant will start the activity program immediately. Gradually, physical activities are replaced by other activities.

### Perpetuating factors: *low social support and negative social interactions*

Low social support and negative social interactions are based on the discrepancy subscale of the Social Support List [49]. If a participant still has unrealistic expectations of others or perceives a discrepancy between actual support and desired support, the therapist helps to install more realistic expectations toward the participant's social support group. The partner or caregiver will be included in this treatment module.

## Pre-publication history

The pre-publication history for this paper can be accessed here:

http://www.biomedcentral.com/1471-2377/10/56/prepub
